# The effects of vitamin D supplementation in carpal tunnel syndrome treatment outcomes: a systematic review

**DOI:** 10.1186/s40634-021-00393-4

**Published:** 2021-09-07

**Authors:** Chirathit Anusitviwat, Porames Suwanno, Sitthiphong Suwannaphisit

**Affiliations:** Department of Orthopedics, Faculty of Medicine, Prince of Songkla University, 15 Karnjanavanich Road, Hat Yai, Songkhla, 90110 Thailand

**Keywords:** Carpal Tunnel Syndrome, Outcome, Vitamin D, Supplementation

## Abstract

**Purpose:**

Vitamin D deficiency is related to carpal tunnel syndrome symptoms. Correcting vitamin D levels by supplementation was supposed to improve carpel tunnel symptoms, though there is a lack of aggregated data about treatment outcomes. This study aimed to examine whether vitamin D supplementation could improve the treatment outcomes in carpal tunnel syndrome patients.

**Methods:**

A comprehensive search of the PubMed, Cochrane Library, Scopus, and Web of Science databases for articles on vitamin D and carpel tunnel syndrome from January 2000 to March 2021 was performed. The article screening and data extraction were performed by two investigators independently with blinding to decisions on selected studies. All included studies had assessed the quality of evidence using the Methodological Index for Non-Randomized Studies (MINORS) scoring system.

**Results:**

We retrieved four studies that met the eligibility criteria. The treatment outcomes were evaluated by visual analog scale (124 wrists), functional scores (176 patients), muscle strength (84 patients), and nerve conduction velocity (216 wrists). After vitamin D supplementation, two studies reported improved pain scores and nerve conduction velocity, and three studies showed enhancement of functional status.

**Conclusion:**

Vitamin D administration could offer favorable outcomes in pain improvement, better functional status, and increased sensory conduction velocity in carpal tunnel syndrome. However, there is to date no recommendations concerning a standardized dose or duration of vitamin D administration in carpal tunnel syndrome; prescribing vitamin D at the usual appropriate dose is suggested as an additional treatment in patients with mild to moderate carpel tunnel symptoms.

**Level of Evidence:**

Level IV, therapeutic study

**Supplementary Information:**

The online version contains supplementary material available at 10.1186/s40634-021-00393-4.

## Introduction

Carpal tunnel syndrome (CTS), peripheral compressive neuropathy of the median nerve in the wrist, is a common disease with a reported uptrend in incidence [5]. Due to compression of the median nerve, CTS patients often experience intractable pain or numbness in the three radial fingers of the hand and/or develop weakness of intrinsic hand muscles, resulting in functional disability [7, 14]. The disease occurs more commonly in females than males, most commonly in adults aged 30–50, and is usually bilateral [1, 2]. There are several known risk factors for CTS, both medical and mechanical [30]. Performing repetitive work using the wrist or handling vibrating instruments for a long time are potential mechanical risk factors. For medical risk factors, hypothyroidism, diabetes mellitus, rheumatoid arthritis, and pregnancy also contribute to developing CTS [1, 19]. In the early stages of CTS, conservative treatment such as oral medications, steroid injections, splinting, therapeutic modalities (ultrasound and iontophoresis), exercise (tendon and nerve gliding), and activity modification [no comma]is the initial approach for alleviating symptoms [6, 31]. However, some patients do not respond well to conservative treatment and may then progress to carpal tunnel release surgery [3].

Recently some studies have reported a potential linkage between CTS and vitamin D levels, and Vitamin D deficiency is recognized as an independent risk factor for increasing severity of CTS symptoms, particularly tingling pain and nerve function [4, 10, 37]. The reason for this connection is that vitamin D, in the form of D2 (ergocalciferol) or D3 (cholecalciferol), has neuroprotection and neurotrophic functions, and also improves nerve myelination which hastens recovery after nerve injuries [8, 9, 27]. Apart from the effect on neurologic function, vitamin D plays a role in suppressing the vascular endothelial growth factor associated with increased inflammatory fibrosis, which may have a role in triggering CTS [16, 17]. And in relation to pain perception, low vitamin D is related to hypersensitivity of nerve fibers leading to persistent painful neuropathy [10, 20].

Eliminating or reducing modifiable risk factors is suggested to reduce the severity of many diseases, including carpel tunnel syndrome. Therefore, correcting low vitamin D levels with vitamin D supplementation is expected to improve treatment outcomes. Previous studies have found that vitamin D levels were correlated with CTS treatment outcomes [29, 32]. However, to date there have been no conclusions or data aggregations concerning the effects of vitamin D supplementation on CTS treatment. The aim of this study was to evaluate earlier studies and summarize whether vitamin D supplementation could improve CTS treatment outcomes.

## Materials and methods

### Search strategy

We conducted this systematic review according to the guidelines of the Preferred Reporting Items for Systematic Reviews and Meta-Analyses (PRISMA) statement [25]. The search strategy was formulated to focus on the outcomes after vitamin D supplementation in CTS patients, using the key terms “vitamin D,” “D2,” “D3,” “hypovitaminosis D,” or “calciferol” combined with “carpal tunnel syndrome,” “CTS,” “median nerve entrapment,” “median neuritis,” “median nerve compression,” or “compressive neuropathy.” Two investigators (CA and SS) searched for published studies indexed in the PubMed, Cochrane Library, Scopus, and Web of Science databases from January 2000 to March 2021 (Additional file 1). After removing duplicate studies, the eligibility of the remaining studies was independently assessed by two investigators (CA and SS) blinded to the other’s decisions on selected studies. Differences were resolved by mutual agreement. If a disagreement persisted, we consulted the third investigator (PS) to arrive at a consensus. Only full articles in English were included.

### Eligibility criteria

All studies which reported an outcome or relation of vitamin D supplementation in CTS patients were reviewed. Original articles written in English were included if they met the following criteria: (1) Cohort (prospective or retrospective) or randomized control trial studies comparing before and after vitamin D replacement or comparing two groups between CTS patients who received vitamin D supplement and who did not, (2) Patients who had a clinical diagnosis of CTS based on symptoms and specific physical findings, or electrodiagnostic studies, (3) Described the dose and details of vitamin D supplementation, (4) Reported data on the outcome of treatment in terms of clinical conditions or laboratory results, and (5) Follow-up duration not less than three months. No restrictions were set regarding the stage of CTS or route of vitamin D administration. We excluded studies with less than 30 participants. Studies which involved patients who had underlying diseases that needed vitamin D as a treatment were also excluded.

### Search results

**Fig. 1:**
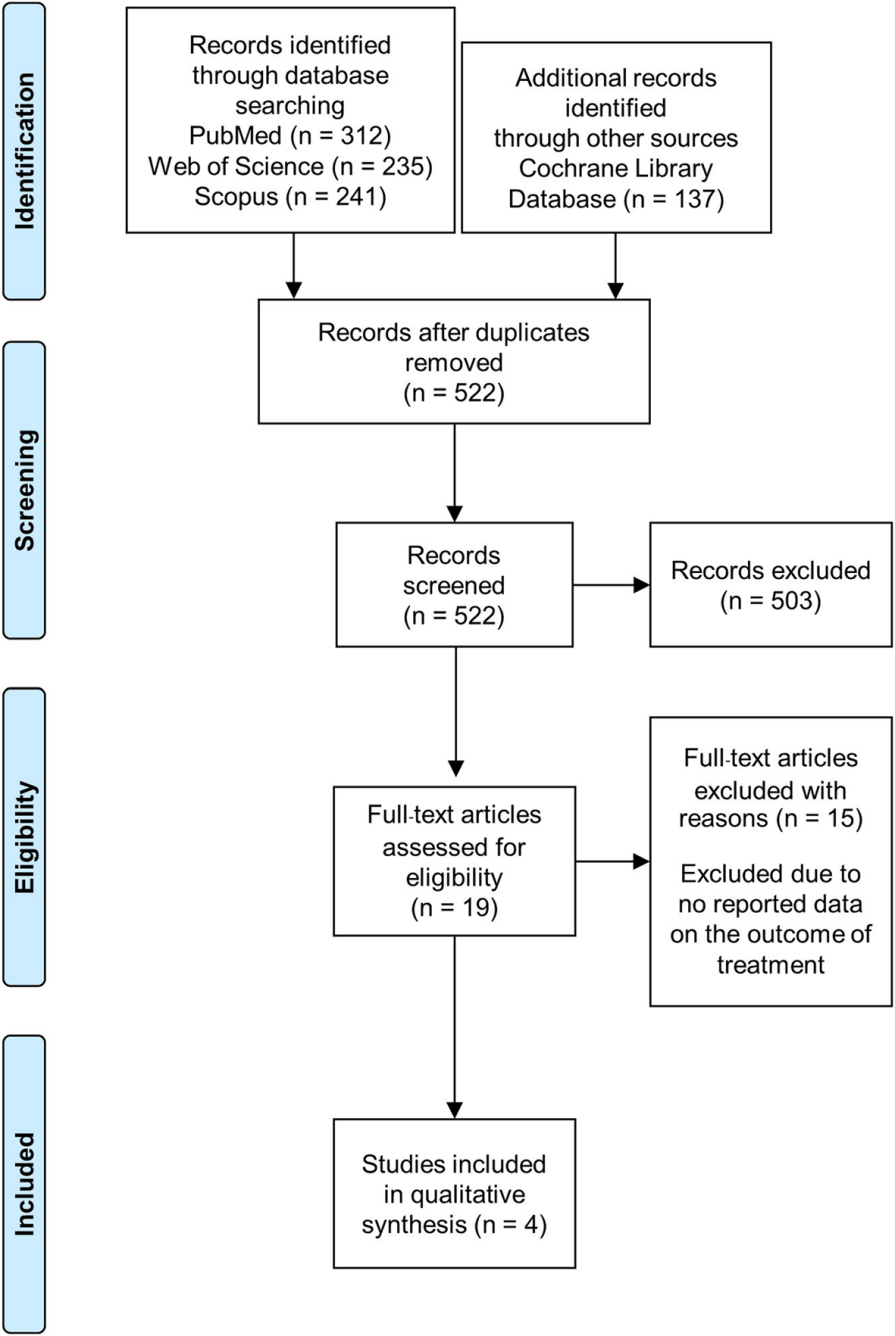
The flowchart of study selection following the Preferred Reporting Items for Systematic Reviews and Meta-Analyses (PRISMA)

From the database search we retrieved a total of 925 studies for evaluation. After removing duplicate results, 522 studies remained and the abstract of each was examined by two investigators (CA and SS). After the abstract reviews 503 studies were excluded as not meeting the inclusion criteria, and another 15 studies were excluded after full-text assessments found no information on the treatment outcomes, leaving four studies for the final analysis (Fig. 1).

### Quality assessment

Two investigators (CA and SS) reviewed and graded each of the four included studies for quality. We applied the Methodological Index for Non-Randomized Studies (MINORS) scoring system to all included studies. Each of the 12 MINORS items is scored 0 to 2, score 0 if not reported, 1 when reported with inadequacy, 2 when reported and adequate. A perfect score was 16 for non-comparative studies and 24 for comparative studies. The quality of each study was categorized according to total scores based on a previous study: 0–5 points indicated poor quality, 6–10 points indicated moderate quality, and 11–16 points indicated good quality [11].

### Data collection and extraction

Duplicate data extraction forms were used for comparison of all outcomes and for achieving completeness with consistency. Any discrepancies were resolved by the senior author (PS). The information in each included study was extracted and recorded. The extracted data about overall study characteristics, patient demographics, including age and gender, and severity of CTS, were evaluated. We also explored the intervention details, including the form and dose of vitamin D along with the duration of administration. Other related CTS treatments and vitamin D levels between before and after intervention were assessed. We extracted CTS symptoms in pain scores, functional scores, muscle strength, and laboratory findings from electrophysiological results to determine vitamin D supplementation outcomes. Any complications after treatment were also evaluated if available.

## Results

### Study characteristics

Of the four studies, two studies each were retrospective and prospective. All included studies were pre-post studies with no control group or randomization. All studies were recently published, not earlier than 2018, and three were conducted in Asia and one in Europe-Asia (Table 1). For our research question about the outcomes after vitamin D supplementation, all of the included studies were rated as moderate quality with the mean MINORS scores across studies 10 of 16 (range, 7–13). The average MINORS scores in the retrospective studies were 8 of 16, while the average scores in the prospective studies were 12 of 16. Scores from three studies were deducted from the prospective calculation of the study size and unbiased assessment of the study endpoint (Additional file 2).

**Table 1:** Characteristics of included studies

First Author	Year of publication	Country	Study design	Recruitment Period	Number of patients	MINORS scores
Samant et al. [36]	2021	India	Prospective cohort	2020	42	11/16
Saçmaci et al. [35]	2020	Turkey	Retrospective cohort	2017–2018	55	9/16
Lee et al. [21]	2019	Korea	Retrospective cohort	2014–2016	84	7/16
Mohtashamkia et al. [26]	2018	Iran	Prospective cohort	2015	50	13/16

*MINORS* Methodological items for non-randomized studies

### Patient demographics and Intervention

The overall number of patients in the four studies was 231, with 258 wrists included for evaluation. The mean age of patients was between 40 to 60 years, with females predominate even considering one report which did not distinguish between genders [26]. Before vitamin supplementation, three studies reported on the severity of CTS as mild or moderate [26, 35, 36]. Previous and ongoing treatment of CTS were analgesics, anti-inflammatory drugs, splint, physiotherapy, and carpal tunnel release [21, 26, 35, 36]. Serum vitamin D levels increased significantly after vitamin supplement in the three studies which reported this [21, 35, 36], whereas one study did not detail the vitamin D level after supplementation [26]. The doses of oral vitamin D varied in each study, ranging from 7000 to 60,000 IU/week with a duration of 12 weeks in three studies [26, 35, 36] and 24 weeks in one study [21]. These clinical data and interventions in each included study are presented in Table 2.

**Table 2:** Clinical and laboratory data of the patients included in each study

First Author	Number of wrists	Gender (M/F)	Mean age (years)	Severity	Serum Vitamin D (ng/ml)		Form and Dose	Duration (weeks)	Other Treatments
					Before	After			
Samant et al. [36]	42	9/33	40.88 ± 7.6	Mild and Moderate CTS	13.73 ± 3.9	46.61 ± 5.8	Oral Vitamin D3; dose 60,000 IU/week	12	Analgesics, Anti-inflammatory drugs, Splint, Physiotherapy
Saçmaci et al. [35]	82	0/55	48.04 ± 6.03 in Mild CTS, 45.97 ± 7.97 in Moderate CTS	45 Mild CTS and 37 Moderate CTS	11.75 ± 4.53 in Mild CTS, 11.64 ± 3.55 in Moderate CTS	39.97 ± 16.02 in Mild CTS, 32.45 ± 13.99 in Moderate CTS	Oral Vitamin D2 or D3; dose 6000 IU/day or 50,000 IU/week	12	Splint
Lee et al. [21]	84	0/84	56 ± 9	Not reported	12 ± 3.6	24.5 ± 8.5	Oral Vitamin D; dose 1000 IU/day	24	Carpal tunnel release
Mohtashamkia et al. [26]	50	Not reported	44 ± 8.5	Mild and Moderate CTS	19.1 ± 6.3	Not reported	Oral Vitamin D3; dose 50,000 IU/week	12	Anti-inflammatory drugs, Splint, Physiotherapy

*CTS* Carpal tunnel syndrome; *IU* International Units

### Outcomes of vitamin D supplementation: Pain, Function, Nerve conduction

The various methods for evaluating CTS symptoms, including pain scores, functional scores, muscle strength, and electrophysiological results, are shown in Table 3. The outcomes before and after the vitamin D supplementation were compared to show the difference in all studies. All studies used combined clinical conditions and nerve conduction velocity to assess the treatment outcomes, except for one study that used only clinical evaluations [36]. The treatment outcomes were evaluated by visual analog scale (VAS) in 124 wrists of 97 patients, functional scores in 176 wrists of 176 patients, muscle strength in 84 wrists of 84 patients, and nerve conduction velocity in 216 wrists of 189 patients. CTS symptoms as evaluated by VAS scores were decreased significantly in two studies [35, 36]. Contrary to the pain scores, there were no statistically significant differences in motor power and median motor conduction velocity. However, median sensory conduction velocity was significantly improved [21, 35]. The functional outcomes were assessed by the Disabilities of the Arm, Shoulder, and Hand (DASH) scores ranging from 0 (no disability) to 100 (most severe disability), the Boston Symptoms Severity Scale (SSS) ranging from 1 to 5 in 11 items, and the Boston Functional Status Scale (FSS) ranging from 1 to 5 in 8 items. Of the four studies, three of them reported a better functional status after vitamin D supplementation [21, 26, 36]. There were no reported complications after the intervention in any of the four studies.

**Table 3:** Outcomes before and after vitamin D supplementation in carpal tunnel syndrome patients

First Author	Evaluation methods	VAS score		Functional score		Nerve conduction velocity		Complications
		Before	After	Before	After	Before	After	
Samant et al. [36]	Functional scores, Symptomatic outcomes	6.42 ± 0.63	3.88 ± 0.94	Boston SSS 2.22 ± 0.43, Boston FSS 2.27 ± 0.37	Boston SSS 1.37 ± 0.20, Boston FSS 1.71 ± 0.36	Not reported	Not reported	None
Saçmaci et al. [35]	Pain scores, Median sensory and motor conduction study	6.64 ± 1.13 in Mild CTS, 7.45 ± 0.69 in Moderate CTS	4.53 ± 0.69 in Mild CTS, 6.64 ± 0.85 in Moderate CTS	Not reported	Not reported	*Sensory CV* 43.67 ± 3.11 in Mild CTS, 37.27 ± 5.46 in Moderate CTS *Motor CV* 55.74 ± 2.88 in Mild CTS, 55.0 ± 2.71 in Moderate CTS	*Sensory CV* 47.25 ± 5.77 in Mild CTS, 40.47 ± 7.31 in Moderate CTS *Motor CV* 55.71 ± 2.79 in Mild CTS, 54.50 ± 3.56 in Moderate CTS	None
Lee et al. [21]	DASH score, Muscle strength, Median motor conduction velocity	Not reported	Not reported	DASH 35, grip strength 16.8 kg, pinch strength 3.7 kg	DASH 17, grip strength 18.6 kg, pinch strength 4.0 kg	*Motor CV* 54.9	*Motor CV* 56	None
Mohtashamkia et al. [26]	Functional scores, Nerve conduction study	Not reported	Not reported	Boston SSS 3.45 ± 0.90, Boston FSS 3.41 ± 0.85	Boston SSS 2.04 ± 0.71, Boston FSS 2.08 ± 0.82	27.02 ± 4.47	32.58 ± 4.54	None

*CTS* Carpal tunnel syndrome; *VAS* visual analog scale; *CV* Conduction velocity; *DASH* Disabilities of the Arm, Shoulder, and Hand; *SSS* Symptoms Severity Scale; *FSS* Functional Status Scale

## Discussion

Some studies in recent years have reported that hypovitaminosis D is one factor that associated with decreased median nerve function [20, 32, 37]. Consequently, some orthopedists have attempted to correct serum vitamin D by prescribing oral vitamin D to improve CTS symptoms. We aggregated recent studies that compared the treatment results between before and after vitamin D supplementation to assess the treatment effect in CTS patients. Although the studies involved only a short period of vitamin D administration and a wide range of vitamin D dosages, all studies reported better outcomes after vitamin supplementation in both clinical and laboratory aspects. These studies showed improved symptoms by reduced VAS pain scores and enhanced hand function as assessed by Boston SSS, Boston FSS, and/or DASH scores. We also found favorable results in nerve conduction velocity studies.

### Pain scores

For patients with low serum vitamin D, vitamin supplementation has been found to effectively relieve pain from compressive neuropathy in CTS [35, 36]. In our systematic review, the pooled VAS scores of 124 wrists reduced from 6.83 ± 0.95 to 5.01 ± 1.44 out of 10 after vitamin D supplementation. These results can be explained by noting that vitamin D plays a vital role in the pain signaling pathway, with supporting in vivo and in vitro studies [15]. Additionally, vitamin D supplementation has been found to have benefits for acute or chronic pain relief in other orthopedic diseases, including growing pain, low back pain, and osteoarthritis [13, 24, 28]. However, the dosage, duration, and type of vitamin D for relieving pain are different in each disease.

### Functional outcomes

In the reviewed studies, the specific functional assessment tool for CTS was the Boston Carpal Tunnel Syndrome test, while the non-specific tool was the DASH test. After 12 weeks of vitamin D supplementation, DASH scores were reduced by 18 percent. Consistent with the DASH scores, the pooled Boston SSS scores reduced from 2.89 ± 0.95 to 1.73 ± 0.64 and the Boston FSS scores reduced from 2.89 ± 0.88 to 1.91 ± 0.68. Our systematic review highlights that those functional scores of CTS patients were improved after vitamin D supplementation regardless of the specificity of the functional assessment tool. However, grip and pinch strength, indirectly reflecting the muscles innervated by the median nerve, were not changed. These different results may have been due to too short period of vitamin D supplementation to affect the motor functions [34]. There is currently no best standard assessment tool for evaluating functional outcomes. For these reasons, the combination of various reliable tools is recommended to optimize the reliability of evaluating functional status in CTS patients [18]. Although there is a lack of study using multiple functional assessment tools with long-term follow-up, the evidence suggests that vitamin D could enhance CTS patients' functional capacity recovery.

### Nerve conduction study

Conduction abnormalities from nerve compression can be tested by nerve conduction velocity studies, assisting clinicians in either diagnosis or evaluating treatment outcomes [33]. These tests are used to evaluate motor and sensory function. Of the three previous studies observing nerve conduction outcomes, only one study reported results from both types of nerve conduction, with details of latency, amplitude, and conduction velocity [35]. Another of the three studies performed electrophysiological evaluations by motor conduction velocity test alone [21], similar to the previous study [20]. The third reported anonymous nerve conduction velocity results without details [26]. Most of the included patients had mild to moderate CTS severity, which affected the median sensory conduction earlier than motor conduction [1]. In all of these studies, the median nerve conduction velocity results significantly improved after vitamin D supplementation [26, 35]; however, the motor conduction velocity was not noticeably changed, which was in line with non-improved grip and pinch strength [21, 35]. Significant enhancement of motor conduction velocity may occur if the CTS severity exceeds mild-moderate symptoms, or with a more extended vitamin D administration period. This systematic review found that vitamin D supplementation altered only median sensory conduction velocity in mild and moderate CTS severity.

### Complications

There were no reported complications following vitamin D administration in this study. Some previous studies reported complications that occurred from iatrogenic accidental overdoses [22]. The clinical presentation of vitamin D toxicity may range from asymptomatic to life-threatening features resulting from persistent, prolonged hypercalcemia. Although prescribing the usual therapeutic dose of vitamin D for a long period may lead to hypercalcemia, another study reported that overall adverse events did not noticeably increase [23]. Therefore, oral vitamin D administration with the appropriate dose is safe for CTS patients.

### Limitations

This review had several limitations. All included studies were cohort studies without a control group, and, according to the methodological index, there were no high-quality studies. Another point about study design is that half of these studies were retrospective, which may have increased the risk of selection and information bias. For these reasons, there could have been a tendency to overestimate the benefits of the intervention. Additionally, only four studies in this systematic review resulted in an overall small number of included patients, even considering the non-restrictive eligibility criteria. And within the small number of patients, they were predominately female, limiting the generalizability of the findings. Regarding the current situation, there are no standardized dosages or forms of vitamin D, whether vitamin D2 or D3, for reducing undesired symptoms in peripheral neuropathy; various vitamin D doses and forms have been recommended [12, 38]. Not only the vitamin D administration but also the concomitant treatments were different in each study, which could have interfered with analysis of the specific effectiveness of vitamin D in CTS. The improved outcomes demonstrated in the studies may have occurred by chance. Finally, these studies had different methods of assessing outcomes. Some used pain scores or functional scores only, whereas some combined these scores with nerve conduction studies. These differences prevented us from directly comparing the outcomes after vitamin D supplementation, which was our primary objective.

More studies are needed to evaluate the influence of vitamin D supplementation in CTS treatment. Future studies should be well-designed, randomized, double-blind trials, minimizing the risk of bias, comparing outcomes between intervention and control groups, together with controlling potential confounders and assessing outcomes by multiple assessment tools. Also, a longer period of follow-up is required to ensure that the reported improvements are long-term without any adverse events.

## Conclusion

The studies in this systematic review showed that vitamin D supplementation at least 12 weeks could offer favorable outcomes in pain improvement, better functionality, and increased sensory conduction velocity for CTS patients with mild-moderate symptoms. However, there was no standardized dose or duration of vitamin D administration in CTS. Based on current evidence, prescribing the usual appropriate dose of oral vitamin D as an additional treatment while monitoring serum vitamin D levels is suggested.

## Supplementary Information

**Additional file 1.**

**Additional file 2.**

## Data Availability

All data generated or analysed during this study are included in this published article and its supplementary information files.
