# Supplementary material for: The effects of vitamin D supplementation in carpal tunnel syndrome treatment outcomes: a systematic review
Source: J Exp Orthop. 2021 Sep 7;8:73. doi: 10.1186/s40634-021-00393-4 (PMC8421488; doi:10.1186/s40634-021-00393-4)
Supplement: Supplementary file 1 — Additional file 1. [file 40634_2021_393_MOESM1_ESM.docx]

**Appendix 1** Search strategies in electronic databases

| Database | Search strategy | Retrieved articles |
| --- | --- | --- |
| PubMed | ("vitamin d"[MeSH Terms] OR "vitamin d"[All Fields] OR "ergocalciferols"[MeSH Terms] OR "ergocalciferols"[All Fields] OR "D3"[All Fields] OR "D2"[All Fields] OR ("ergocalciferols"[MeSH Terms] OR "ergocalciferols"[All Fields] OR "calciferol"[All Fields] OR "calciferols"[All Fields]) OR ("avitaminosis"[MeSH Terms] OR "avitaminosis"[All Fields] OR "hypovitaminosis"[All Fields])) AND ("carpal tunnel syndrome"[MeSH Terms] OR ("carpal"[All Fields] AND "tunnel"[All Fields] AND "syndrome"[All Fields]) OR "carpal tunnel syndrome"[All Fields] OR ("clin transl sci"[Journal] OR "cts"[All Fields]) OR (("compress"[All Fields] OR "compressed"[All Fields] OR "compresses"[All Fields] OR "compressibilities"[All Fields] OR "compressibility"[All Fields] OR "compressible"[All Fields] OR "compressing"[All Fields] OR "compression"[All Fields] OR "compression s"[All Fields] OR "compressions"[All Fields] OR "compressive"[All Fields] OR "compressively"[All Fields]) AND ("neuropathies"[All Fields] OR "neuropathy"[All Fields])) OR ("median nerve"[MeSH Terms] OR ("median"[All Fields] AND "nerve"[All Fields]) OR "median nerve"[All Fields]) OR ("nerve compression syndromes"[MeSH Terms] OR ("nerve"[All Fields] AND "compression"[All Fields] AND "syndromes"[All Fields]) OR "nerve compression syndromes"[All Fields] OR ("nerve"[All Fields] AND "entrapment"[All Fields]) OR "nerve entrapment"[All Fields]) OR (("nerve"[All Fields] OR "nerve s"[All Fields] OR "nerved"[All Fields] OR "nerves"[All Fields]) AND ("compress"[All Fields] OR "compressed"[All Fields] OR "compresses"[All Fields] OR "compressibilities"[All Fields] OR "compressibility"[All Fields] OR "compressible"[All Fields] OR "compressing"[All Fields] OR "compression"[All Fields] OR "compression s"[All Fields] OR "compressions"[All Fields] OR "compressive"[All Fields] OR "compressively"[All Fields])) OR (("median"[All Fields] OR "medians"[All Fields]) AND ("neuritis"[MeSH Terms] OR "neuritis"[All Fields] OR "neuritides"[All Fields] OR "polyneuritides"[All Fields]))) | 312 |
| Cochrane Library | (((((vitamin D) OR D3) OR D2) OR calciferol) OR hypovitaminosis) AND (((((((carpal tunnel syndrome) OR CTS) OR compressive neuropathy) OR median nerve) OR nerve entrapment) OR nerve compression) OR median neuritis) | 137 |
| Scopus | ALL ( ( ( ( ( ( vitamin AND d ) OR d3 ) OR d2 ) OR calciferol ) OR hypovitaminosis ) AND ( ( ( ( ( ( ( carpal AND tunnel AND syndrome ) OR cts ) OR compressive AND neuropathy ) OR median AND nerve ) OR nerve AND entrapment ) OR nerve AND compression ) OR median AND neuritis ) ) AND ( LIMIT-TO ( LANGUAGE , "English" ) ) | 241 |
| Web of Science | (ALL= ((vitamin D OR D3 OR D2 OR calciferol OR hypovitaminosis) AND (carpal tunnel syndrome OR CTS OR compressive neuropathy OR median nerve OR nerve entrapment OR nerve compression OR median neuritis) )) AND LANGUAGE: (English) Indexes=SCI-EXPANDED, SSCI, A&HCI, ESCI Timespan=All years | 235 |
