# Supplementary material for: The effects of vitamin D supplementation in carpal tunnel syndrome treatment outcomes: a systematic review
Source: J Exp Orthop. 2021 Sep 7;8:73. doi: 10.1186/s40634-021-00393-4 (PMC8421488; doi:10.1186/s40634-021-00393-4)
Supplement: Supplementary file 2 — Additional file 2. [file 40634_2021_393_MOESM2_ESM.docx]

| Included Studies | Methodological items for non-randomized studies | | | | | | | | | | | | | |
| --- | --- | --- | --- | --- | --- | --- | --- | --- | --- | --- | --- | --- | --- | --- |
|  | A clearly stated aim | Inclusion of consecutive patients | Prospective collection of data | Endpoints appropriate to the aim of the study | Unbiased assessment of the study endpoint | Follow-up period appropriate to the aim of the study | Loss to follow up less than 5% | Prospective calculation of the study size | An adequate control group | Contemporary groups | Baseline equivalence of groups | Adequate statistical analyses | Total |  |
| Samant et al.[36] | 2 | 2 | 2 | 1 | 0 | 2 | 2 | 0 | NA | NA | NA | NA | 11/16 |  |
| Saçmaci et al.[35] | 2 | 2 | 0 | 1 | 0 | 2 | 2 | 0 | NA | NA | NA | NA | 9/16 |  |
| Lee et al.[21] | 2 | 0 | 0 | 1 | 0 | 2 | 2 | 0 | NA | NA | NA | NA | 7/16 |  |
| Mohtashamkia et al.[26] | 2 | 2 | 2 | 1 | 2 | 2 | 2 | 0 | NA | NA | NA | NA | 13/16 |  |

**Appendix 2** Methodological items for non-randomized study scores for outcome of vitamin D supplementation

*NA* not applicable
